# Prasugrel for Japanese patients with acute coronary syndrome in short-term clinical practice (PRASFIT-Practice I): a postmarketing observational study

**DOI:** 10.1007/s12928-017-0459-8

**Published:** 2017-02-17

**Authors:** Masato Nakamura, Tomoko Iizuka, Kei Sagawa, Kenji Abe, Shuichi Chikada, Miyuki Arai

**Affiliations:** 1 Division of Cardiovascular Medicine, Toho University Ohashi Medical Center, 2-17-6 Ohashi, Meguroku, Tokyo 153-8515 Japan; 2 Pharmacovigilance Department, Daiichi Sankyo Co., Ltd., Tokyo, Japan; 3 Safety and Risk Management Department, Daiichi Sankyo Co., Ltd., Tokyo, Japan; 4 Biostatistics and Data Management Department, Daiichi Sankyo Co., Ltd., Tokyo, Japan

**Keywords:** Acute coronary syndrome, Bleeding adverse events, Percutaneous coronary intervention, Postmarketing observational study, Prasugrel

## Abstract

Data on prasugrel use in Japanese patients are limited to phase II/III clinical trials. This early postmarketing observational study evaluated the safety and efficacy of short-term prasugrel use in patients with acute coronary syndrome (ACS) in real-world clinical settings in Japan. From May 2014 to January 2015, we enrolled consecutive patients with ACS requiring percutaneous coronary intervention in each institution. Each patient started prasugrel treatment ≥1 month before the end of the study period. Safety outcomes included incidence rates of adverse drug reactions (ADRs) and bleeding adverse events (AEs). Efficacy outcomes were incidence rates of cardiovascular events (including major adverse cardiovascular events [MACE]). Case report forms were collected from 749 patients, 732 of whom were eligible for the safety and efficacy analysis sets. Approximately 95% of patients had a prasugrel loading/maintenance dose of 20 mg/3.75 mg/day. The incidences of ADRs and bleeding AEs were 8.6 and 6.4%, respectively. Twelve patients experienced major bleeding AEs; approximately 60% (seven patients) of which were gastrointestinal disorders. The incidence of bleeding AEs was significantly higher primarily in patients of female sex, aged ≥75 years, with low body weight (≤50 kg), severe cardiovascular disease, or severe renal impairment. The incidence of MACE was 1.9% during prasugrel treatment, and 3.1% at the end of the study period. This short-term study indicated that prasugrel treatment at loading/maintenance doses of 20 mg/3.75 mg/day was safe and effective in Japanese ACS patients in an acute setting.

Clinical Trial Registration: This study is registered at http://www.umin.ac.jp/ctr/ under the identifier UMIN000014699.

**Electronic supplementary material:**

The online version of this article (doi:10.1007/s12928-017-0459-8) contains supplementary material, which is available to authorized users.

## Introduction

To avoid complications after percutaneous coronary intervention (PCI), such as acute and late thrombosis at the site of stenting and recurrent ischemic cardiovascular events, anti-platelet therapy is crucial [1]. Several studies [2–4] have shown that dual anti-platelet therapy with aspirin and a thienopyridine ADP-receptor blocker is effective in preventing such acute and late complications. Clopidogrel is an ADP-receptor blocker that is used regularly in Japan. It has a better safety profile than ticlopidine, a previous-generation ADP-receptor blocker, but its main disadvantage is the wide interindividual variations of its anti-platelet effect [5]. Hoshino et al. evaluated the anti-platelet effect of clopidogrel in Japanese patients and found wide interindividual variation as well as a proportion (approximately 14%) of clopidogrel non-responders [6].

Prasugrel is a next-generation thienopyridine anti-platelet agent that has been approved in over 80 countries for patients with acute coronary syndrome (ACS) undergoing PCI. Prasugrel provides more prompt, potent, and consistent platelet inhibition than clopidogrel, and the effects of prasugrel are not influenced by the presence of CYP2C19 polymorphisms [7]. The efficacy and safety of prasugrel were confirmed in Japanese patients in two phase III studies [8, 9]. Based on these results, prasugrel was approved in Japan in March 2014 for ACS [including unstable angina, non-ST-segment elevation myocardial infarction (NSTEMI), and ST-segment elevation myocardial infarction (STEMI)], stable angina, and old myocardial infarction that requires PCI. The approved doses of prasugrel, which are exclusive for Japanese patients, are 20 mg as the initial loading dose (LD) and 3.75 mg/day as the maintenance dose (MD), which are lower than those used in Western countries (LD/MD: 60/10 mg/day). Given the higher average age and lower body weight of Japanese patients compared with Western patients, we considered that lower doses of prasugrel may effectively lower the risk of bleeding during dual anti-platelet therapy in Japanese patients while maintaining more consistent platelet inhibition than clopidogrel.

At present, data on the safety and efficacy of prasugrel in Japanese patients are limited to the populations of the phase II/III clinical trials, in which patients were selected based on strict inclusion criteria. In addition, because bleeding adverse events (AEs) have been reported as the most common adverse drug reactions (ADRs) in clinical trials, safety information should be made available in clinical settings as soon as possible by determining the incidence and severity of bleeding AEs under actual conditions of use. Therefore, this early postmarketing observational study (PRASFIT-Practice I) aimed to evaluate the safety and efficacy of the short-term use of prasugrel in patients with ACS in real-world clinical practice settings in Japan.

## Methods

### Study method

Briefly, this study was conducted as a postmarketing observational study in accordance with the Good Postmarketing Study Practice Guideline (Ministry of Health, Labour and Welfare Ordinance No. 171). At each institution, consecutive patients who met the inclusion criteria were enrolled prospectively. To gather as much information as possible in the period immediately after launch, retrospective data were collected for patients treated with prasugrel before the conclusion of the contract with each institution.

All 98 participating institutions approved the study protocol. Case report forms (CRFs) were collected for each patient who started treatment with prasugrel at least 1 month before the end of the study period, between 27 May 2014 and 26 January 2015. Because all patients were to be followed up until the end of the study period (26 January 2015) regardless of completion or discontinuation of prasugrel treatment, the observation period varied for each patient. Patients with ACS who were to undergo or had recently undergone PCI and had started prasugrel treatment at least 1 month before the end of the study period were included in this study.

### Dosage and administration

Dosage and administration according to the Japanese prescription label of prasugrel are as follows: prasugrel should be initiated with a single 20-mg oral dose and then continued at a 3.75-mg once-daily oral dose as a maintenance dose [10]. Prasugrel was administered as 3.75- and 5-mg tablets, in combination with aspirin (81–100 mg/day; up to 324 mg could be used as an LD). Patients receiving a prasugrel dose of 3.75 mg during approximately 5 days prior to PCI did not require an initial LD. The extent of the exposure to prasugrel and the timing of prasugrel administration (before, during, or after PCI) under the actual conditions of use were examined.

### Study variables

Patient demographics, clinical baseline characteristics, clinical findings assessed prior to prasugrel treatment (or before initial PCI), during hospitalization, and at discharge, and the final diagnosis of ACS were assessed. The extent of exposure to prasugrel was based on the time and date of administration of the LD, the MD, the daily dose, the duration of treatment, and continuation or discontinuation of treatment. Other variables assessed were use of other anti-platelet agents/anti-coagulants and other concomitant medications, invasive procedures other than PCI/coronary artery bypass graft, initial coronary angiography (CAG) findings if the patient underwent CAG, timing of prasugrel administration (before, during, or after PCI), vital signs, laboratory data, AEs, bleeding AEs, and cardiovascular events.

### Safety and efficacy

The safety outcomes assessed were the incidence of ADRs, serious ADRs, and bleeding AEs. The incidence of bleeding AEs was also assessed by clinical characteristics. AEs were defined as any unfavorable or unintended sign (including an abnormal laboratory finding), symptom, or disease showing a temporal association with the use of the study drug, irrespective of whether it was considered to be related to the drug. ADRs were defined as AEs for which a relationship to prasugrel could not be ruled out. AEs or ADRs that satisfied the following criteria were classified as serious: an event which (1) results in death, (2) is life-threatening, (3) requires hospitalization or prolongation of hospitalization, (4) results in disability or significant incapacity, (5) has the potential to result in disability or significant incapacity, (6) is as serious as any of the outcomes listed above, or (7) causes a congenital anomaly or birth defect. Detailed definitions of bleeding and cardiovascular events are provided in Electronic Supplement 1. Regarding the incidence of bleeding AEs by clinical characteristics, we identified clinical characteristics potentially affecting the incidence of bleeding AEs by comparing patients with bleeding AEs with those without.

The efficacy outcomes were the incidence of major adverse cardiovascular events (MACE). MACE was defined as a composite of cardiovascular death, non-fatal myocardial infarction (MI), and non-fatal ischemic stroke. All-cause death, non-fatal stroke, readmission due to angina pectoris, urgent revascularization, and stent thrombosis (defined as definite or probable according to the Academic Research Consortium) were also assessed.

### Statistical analysis

The planned sample size was 500 patients based on the estimated number of patients who were anticipated to receive treatment with prasugrel and on enrollment feasibility. We enrolled consecutive patients in each institution to avoid patient selection bias. For each of the safety and efficacy variables, a point estimate and its 95% confidence interval (CI) were calculated.

The Chi-square test was used for subgroup analyses to identify clinical characteristics potentially affecting the incidence of bleeding AEs. The significance level was set to *α* = 0.05 (two-sided). All statistical analyses were performed with SAS 9.2 (SAS Institute Inc., Cary, NC, USA).

## Results

### Patient disposition and baseline demographic and clinical characteristics

**Fig. 1:**
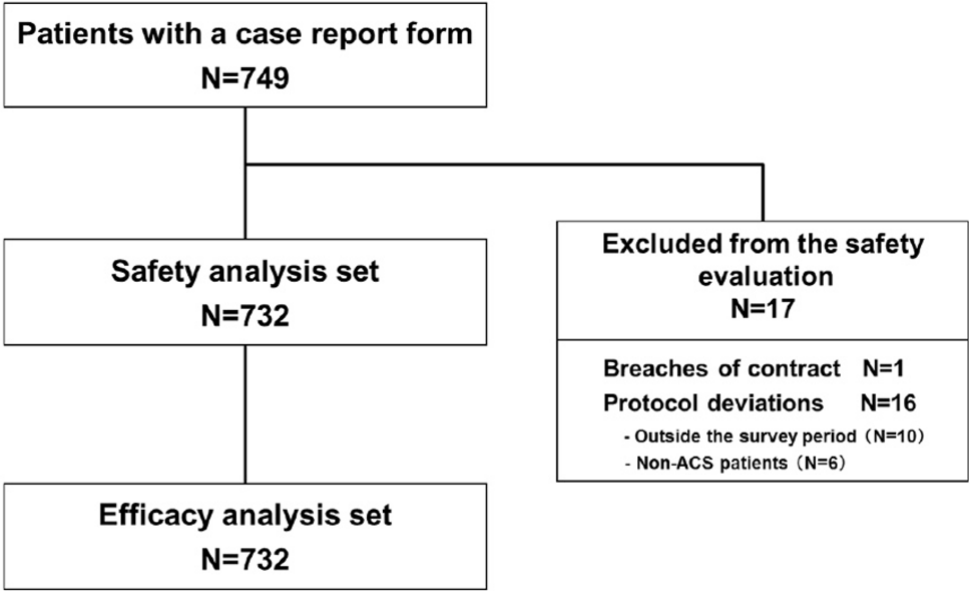
Patient disposition. *ACS* acute coronary syndrome

CRFs were collected from 749 patients at 98 institutions nationwide (Fig. 1). Of these, 732 patients were included in the safety analysis set, excluding those who fell under “breaches of contract” and “protocol deviations”. All 732 patients were included in the efficacy analysis set. The mean (±standard deviation) observation period was 64.9 ± 73.8 days [median (range) 31.0 (1–531) days], regardless of continuation or discontinuation of prasugrel treatment.

Baseline demographic and clinical characteristics of patients are shown in Table 1. In the study population, 60.0% of patients had STEMI. Furthermore, 6.7% of patients had severe cardiovascular disease, classified as Killip Class IV. These patients were excluded from PRASFIT-ACS [8], a phase III clinical trial conducted in Japanese patients with ACS.

**Table 1:** Baseline demographic and clinical characteristics of patients

	PRASFIT-Practice I *N* (%) (*N* = 732)	[Reference] PRASFIT-ACS *N* (%) (*N* = 685)
Sex		
Male	560 (76.5)	536 (78.2)
Age (years)		
≥75	221 (30.2)	165 (24.1)
Mean ± SD	67.0 ± 12.4	65.4 ± 11.4
Median (range)	67 (29–97)	65 (32–95)
Body weight (kg)		
≤50	94 (12.8)	85 (12.4)
Mean ± SD	63.8 ± 12.5	64.2 ± 12.3
Final diagnosis		
STEMI	439 (60.0)	340 (49.6)
NSTEMI	92 (12.6)	187 (27.3)
Unstable angina	198 (27.0)	156 (22.8)
Killip classification		
Class I	572 (78.1)	NA
Class II	91 (12.4)	
Class III	15 (2.0)	
Class IV	49 (6.7)	Exclusion criteria
Medical history		
Prior MI	71 (9.7)	34 (5.0)
Prior revascularizations	92 (12.6)	40 (5.8)
Prior CABG	7 (1.0)	6 (0.9)
Prior TLR	33 (4.5)	15 (2.2)
Prior ischemic stroke	37 (5.1)	Exclusion criteria
Complications		
Hypertension	559 (76.4)	495 (72.3)
Dyslipidemia	565 (77.2)	516 (75.3)
Diabetes mellitus	267 (36.5)	250 (36.5)
History of smoking	249 (34.0)	273 (39.9)
On dialysis	15 (2.0)	Exclusion criteria
Antithrombotic agent		
Prasugrel + aspirin	678 (92.6)	685 (100.0)
Prasugrel + aspirin + WF or DOAC	19 (2.6)	Exclusion criteria
Prasugrel + NSAIDs (w/o aspirin)	11 (1.5)	Exclusion criteria
Concomitant drug		
PPIs	347 (47.4)	282 (41.2)
Stent type		
Drug-eluting stent	671 (91.7)	291 (42.5)
Puncture site		
Brachial	23 (3.1)	22 (3.2)
Radial	374 (51.1)	285 (41.6)
Femoral	315 (43.0)	366 (53.4)

*SD* standard deviation, *STEMI* ST-segment elevation myocardial infarction, *NSTEMI* non-ST-segment elevation myocardial infarction, *MI* myocardial infarction, *CABG* coronary artery bypass graft, *TLR* target lesion revascularization, *WF* warfarin, *DOAC* direct oral anti-coagulant, *NSAIDs* non-steroidal anti-inflammatory drugs, *PPIs* proton pump inhibitors, *NA* not available

Regarding other clinical characteristics that were excluded from the PRASFIT-ACS [8], 5.1% of patients had a history of ischemic stroke; 2.0% were on dialysis; 2.6% were concomitantly using warfarin or direct oral anti-coagulants (DOACs); and 1.5% were using non-steroidal anti-inflammatory drugs (NSAIDs). The radial puncture site was the most common in the present observational study, though the femoral puncture site was the most common in PRASFIT-ACS [11].

### Treatment status of prasugrel and discontinuations

**Fig. 2:**
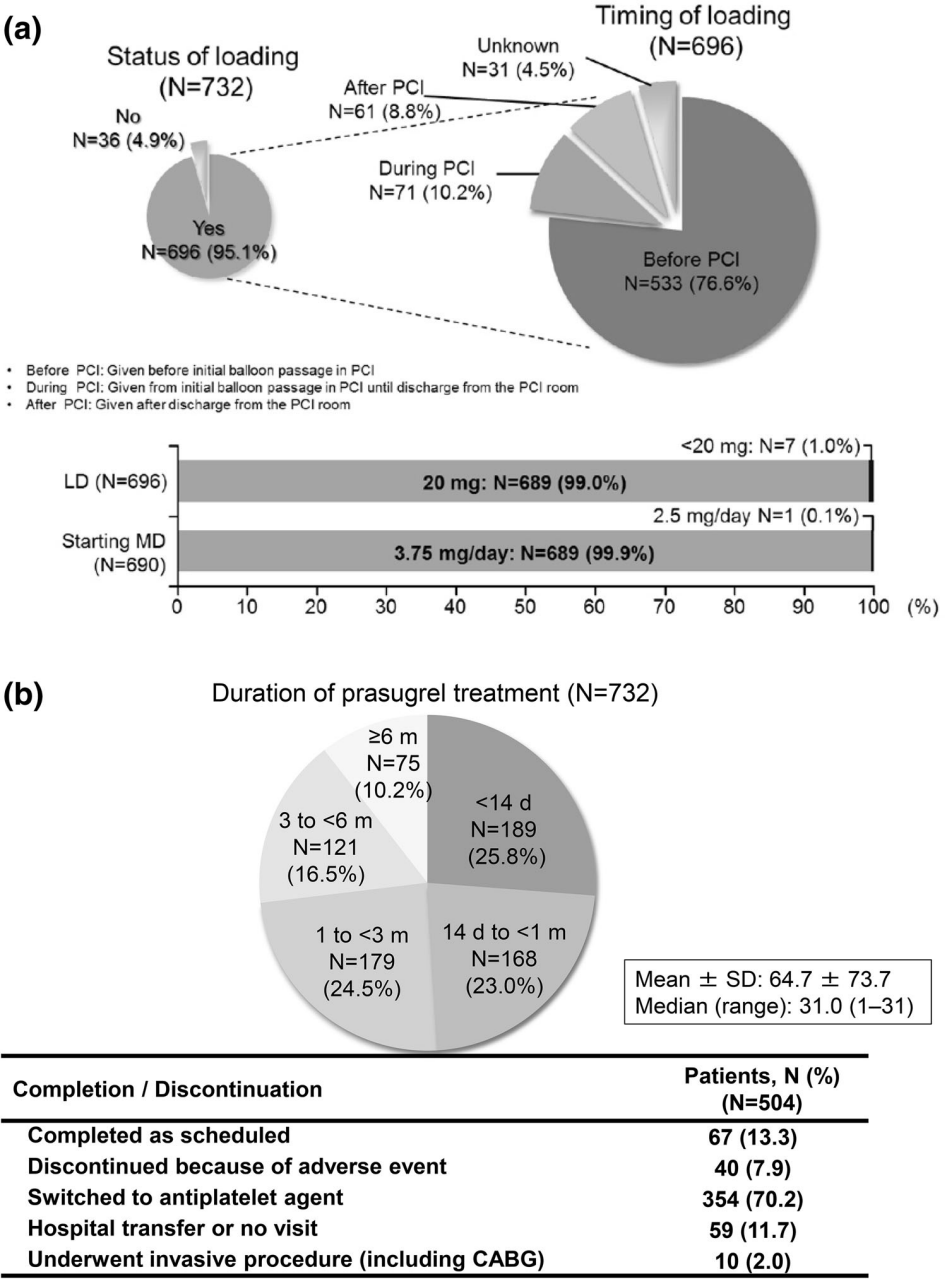
**a** Timing of loading, loading dose (LD), and starting maintenance dose (MD); **b** duration of prasugrel treatment and reasons for discontinuation. *PCI* percutaneous coronary intervention, *CABG* coronary artery bypass grafting

Treatment status of prasugrel and discontinuations are shown in Fig. 2. An initial LD was administered to 95.1% of patients. In the majority of patients, the LD was given before the initial PCI. In 99.0% of patients, the initial prasugrel LD was 20 mg. One out of 690 (0.1%) patients was given an MD of 2.5 mg/day; the remaining patients received an MD of 3.75 mg once-daily.

Almost one-half of the patients completed or discontinued the treatment within 1 month. The most common reason for discontinuation was switching to other anti-platelet agents (70.2%, 354/504). Of 504 patients, 40 (7.9%) discontinued prasugrel treatment because of AEs.

### Safety and efficacy

#### Safety

The incidence of ADRs was 8.6% (63/732); serious ADRs, 3.4% (25/732); and bleeding AEs, 6.4% (Electronic Supplement 2). The most common ADRs were gastrointestinal disorders (e.g., gastrointestinal hemorrhage) [3.3% (24/732)]. The most common serious ADRs were also gastrointestinal disorders [2.0% (15/732)]. Table 2 summarizes the breakdown of bleeding AEs. Bleeding AEs occurred in 6.4% of patients. The most common bleeding AE was gastrointestinal disorders (2.7%), followed by general disorders and administration site conditions (1.0%). Regarding puncture site bleeding (puncture site hemorrhage or vessel puncture site hematoma), the puncture site locations were femoral (three patients), radial (two patients), femoral + radial (one patient), and brachial (one patient). The incidence of major bleeding [thrombolysis in myocardial infarction (TIMI) criteria] AEs was 1.6%. Approximately 60% (7/12) of all major bleeding AEs were gastrointestinal disorders.

**Table 2:** Incidence of bleeding adverse events by severity and site

Item	Result				
No. of patients in the safety analysis set	732				
No. of patients with bleeding AEs	47				
No. of bleeding AEs	52				
Incidence of patients with bleeding AEs (%)	6.4				
No. of patients with major bleeding AEs	12				
Incidence of patients with major bleeding AEs (%)	1.6				

Type of bleeding AE	No. of patients with bleeding AEs; No. of bleeding AEs^a^ (%)	Classification of bleeding (TIMI)			
		Major bleeding (*N* = 12)	Minor bleeding (*N* = 15)	Clinically relevant (*N* = 12)	Other (*N* = 10)
Blood and lymphatic system disorders	5 (0.7)	1	3	–	1
Anemia	5 [2] (0.7)	1	3	–	1
Eye disorders	1 (0.1)	–	–	–	1
Conjunctiva hemorrhage	1 [1] (0.1)	–	–	–	1
Cardiac disorder	3 (0.4)	–	–	3	–
Cardiac tamponade	1 [1] (0.1)	–	–	1	–
Myocardial hemorrhage	2 [2] (0.3)	–	–	2	–
Vascular disorders	2 (0.3)	–	1	–	1
Hematoma	1 [1] (0.1)	–	–	–	1
Bleeding	1 (0.1)	–	1	–	–
Respiratory, thoracic and mediastinal disorders	3 (0.4)	1		2	
Epistaxis	1 (0.1)	–	–	1	–
Hemoptysis	1 (0.1)	–	–	1	–
Pulmonary hemorrhage	1 [1] (0.1)	1	–	–	–
Gastrointestinal disorders	20 (2.7)	7	6	6	2
Hemorrhagic intestinal diverticulum	1 (0.1)	–	–	1	–
Gastrointestinal hemorrhage	6 [6] (0.8)	3	1	2	–
Gingival bleeding	1 (0.1)	–	–	–	1
Hematemesis	1 (0.1)	–	1	–	–
Hematochezia	1 (0.1)	–	–	1	–
Mallory-Weiss syndrome	1 (0.1)	–	1	–	–
Melena	4 [2] (0.5)	–	1	2	1
Rectal hemorrhage	2 [2] (0.3)	2	–	–	–
Upper gastrointestinal hemorrhage	1 [1] (0.1)	1	–	–	–
Large intestinal hemorrhage	1 [1] (0.1)	–	1	–	–
Duodenal hemorrhage	1 [1] (0.1)	1	–	–	–
Hemorrhoidal bleeding	1 (0.1)	–	1	–	–
Skin and subcutaneous tissue disorders	4 (0.5)	–	4	–	–
Subcutaneous hemorrhage	4 (0.5)	–	4	–	–
Renal and urinary disorders	3 (0.4)	1	1	1	–
Hematuria	3 [1] (0.4)	1	1	1	–
General disorders and administration site conditions	7 (1.0)	2	2	–	3
Puncture site hemorrhage	6 [1] (0.8)	1	2	–	3
Vessel puncture site hematoma	1 (0.1)	1	–	–	–
Injury, poisoning and procedural complications	3 (0.4)	–	–	–	3
Subcutaneous hematoma	2 (0.3)	–	–	–	2
Wounds	1 (0.1)	–	–	–	1

For SOC, the number of patients with bleeding AEs was tabulated, and for preferred term, the number of bleeding AEs (i.e., the number of patients for each preferred term) was tabulated. MedDRA/J version 18.1

*AEs* adverse events, *SOC* system organ class, *TIMI* thrombolysis in myocardial infarction

^a^The number of patients for SOC and the number of bleeding AEs for each preferred term were tabulated. The number of serious bleeding AEs is specified in square brackets in the applicable cells

The incidence of bleeding AEs by clinical characteristics is shown in Table 3. The incidence of bleeding AEs was significantly higher in female patients, patients aged 75 years or older, patients with low body weight (50 kg or less), patients with severe cardiovascular disease (Killip Class III or IV), patients without dyslipidemia, and patients with severe renal impairment (creatinine clearance less than 30 mL/min). The proportion of female patients weighing 50 kg or less who experienced bleeding AEs was 40.9% (9/22), compared with 16.0% (4/25) among male patients with bleeding AEs weighing 50 kg or less. Furthermore, puncture site hemorrhage and subcutaneous hemorrhage were reported more frequently in female patients [2.9% (5/172) and 1.7% (3/172), respectively], than in males [0.2% (1/560) and 0.2% (1/560)]. In contrast, variations in timing of the initial LD (before, during, or after PCI) did not significantly affect the occurrence of bleeding AEs.

**Table 3:** Incidence of bleeding adverse events by clinical characteristic

	Patients, *N*	Patients with bleeding AEs, *N* (%)	*P* value*
Safety analysis set	732	47 (6.4)	–
Sex			
Male	560	25 (4.5)	<0.0001
Female	172	22 (12.8)	
Age (years)			
<75	511	22 (4.3)	0.0004
≥75	221	25 (11.3)	
Body weight (kg)^a^			
≤50	94	13 (13.8)	0.0008
>50	610	30 (4.9)	
Final diagnosis^a^			
STEMI	439	33 (7.5)	0.4138
NSTEMI	92	3 (3.3)	
UAP	198	11 (5.6)	
Killip classification^a^			
Class I	572	29 (5.1)	0.0302
Class II	91	8 (8.8)	
Class III	15	2 (13.3)	
Class IV	49	7 (14.3)	
Prior MI^a^			
Absent	653	44 (6.7)	0.1983
Present	71	2 (2.8)	
Prior revascularizations^a^			
Absent	634	43 (6.8)	0.1951
Present	92	3 (3.3)	
Prior CABG			
Absent	725	47 (6.5)	0.4862
Present	7	0 (0.0)	
Prior TLR			
Absent	699	45 (6.4)	0.9312
Present	33	2 (6.1)	
Prior ischemic stroke^a^			
Absent	688	45 (6.5)	0.3508
Present	37	1 (2.7)	
Hypertension			
Absent	173	11 (6.4)	0.9694
Present	559	36 (6.4)	
Dyslipidemia			
Absent	167	20 (12.0)	0.0009
Present	565	27 (4.8)	
Diabetes mellitus			
Absent	465	28 (6.0)	0.5609
Present	267	19 (7.1)	
History of smoking^a^			
Absent	462	36 (7.8)	0.0841
Present	249	11 (4.4)	
Baseline Ccr^a^ (mL/min)			
Normal (>80)	299	9 (3.0)	0.0043
Mild (>50 to ≤80)	238	18 (7.6)	
Moderate (≥30 to ≤50)	97	9 (9.3)	
Severe (<30)	48	7 (14.6)	
Timing of loading^a^			
Before PCI^b^	533	37 (6.9)	0.5973
During PCI^c^	71	3 (4.2)	
After PCI^d^	61	3 (4.9)	
Prasugrel + aspirin			
Not used	54	3 (5.6)	0.7875
Used	678	44 (6.5)	
Prasugrel + aspirin + WF or DOAC			
Not used	713	45 (6.3)	0.4595
Used	19	2 (10.5)	
Prasugrel + NSAIDs (w/o aspirin)			
Not used	721	47 (6.5)	0.3814
Used	11	0 (0.0)	
PPIs			
Not used	385	23 (6.0)	0.6035
Used	347	24 (6.9)	

*NSTEMI* non-ST-segment elevation myocardial infarction, *STEMI* ST-segment elevation myocardial infarction, *UAP* unstable angina pectoris, *MI* myocardial infarction; *CABG* coronary artery bypass graft, *TLR* target lesion revascularization, *Ccr* creatinine clearance, *WF* warfarin, *DOAC* direct oral anti-coagulant, *NSAIDs* non-steroidal anti-inflammatory drugs, *PPIs* proton pump inhibitors, *PCI* percutaneous coronary intervention

* *χ* ^2^ test

^a^Body weight, Killip class, prior MI, prior revascularization, prior ischemic stroke, history of smoking, and timing of loading dose were unknown in 28, 5, 8, 6, 7, 21, and 31 patients, respectively. Three patients had a final diagnosis other than STEMI, NSTEMI, or UAP. Baseline Ccr was not calculated in 50 patients

^b^Given before the initial balloon passage in PCI

^c^Given from the initial balloon passage in PCI until discharge from the PCI room

^d^Given after discharge from the PCI room

**Fig. 3:**
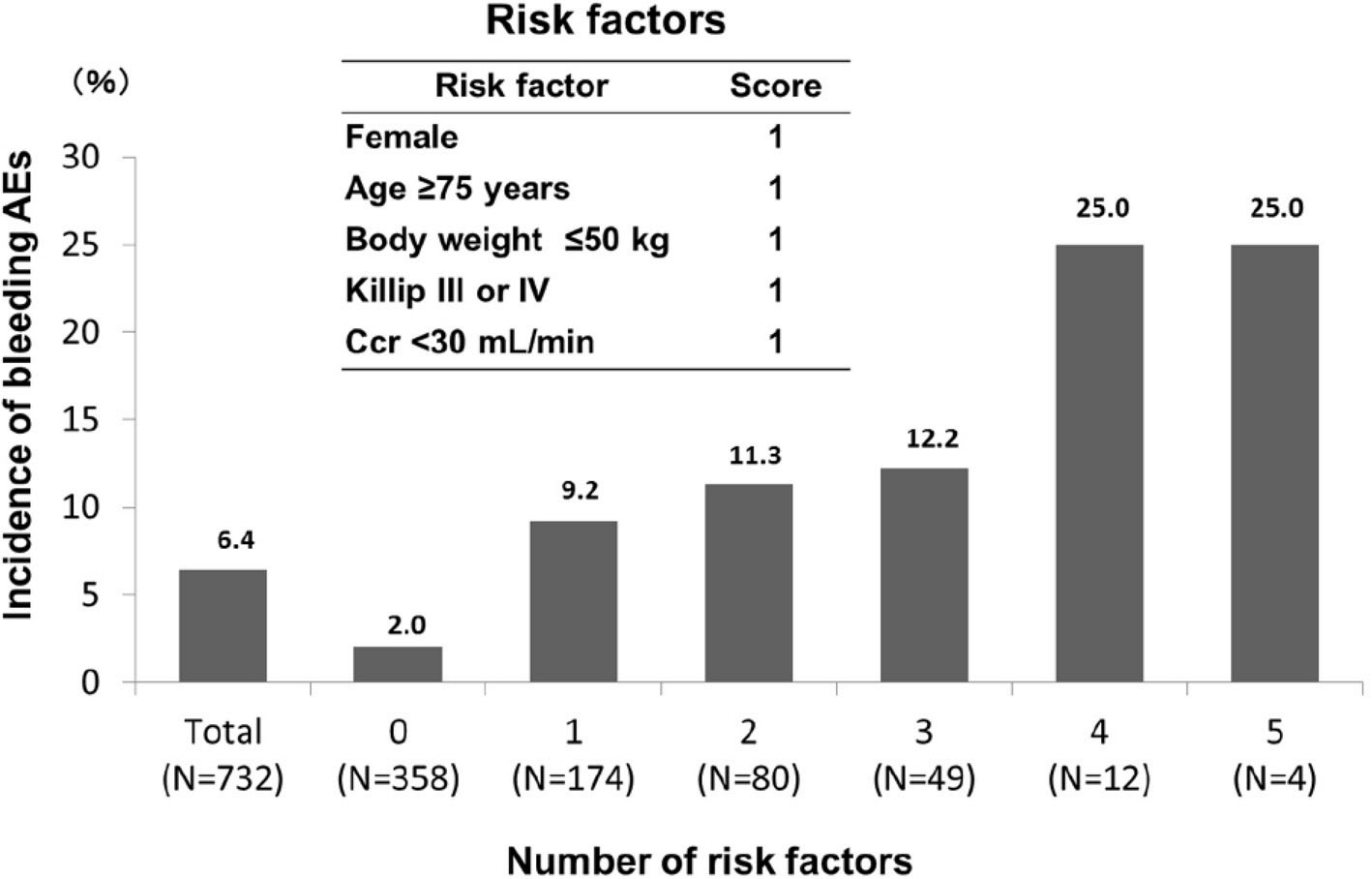
Bleeding adverse events by number of risk factors. *AEs* adverse events, *Ccr* creatinine clearance. *The score of the risk factors was not calculated in 55 patients

Of the above risk factors, each score for the following five main risk factors [female sex, age of 75 years or older, low body weight (50 kg or less), severe cardiovascular disease (Killip Class III or IV), and severe renal impairment (creatinine clearance less than 30 mL/min)] is defined as 1. We calculated the total risk score and the incidence of bleeding AEs. By this analysis (Fig. 3), we found that the bleeding risk increased sharply in patients who had four or all five risk factors.

#### Efficacy

The details of the efficacy analysis are shown in Table 4. The incidence of MACE in the efficacy analysis was 1.9% during prasugrel treatment, and 3.1% up to the end of the observation period. Cardiovascular death was the most common MACE, occurring in 13 patients with an incidence of 1.8% at the end of the study period. Of these, eight patients had severe cardiovascular disease (Killip Class IV). The incidence of all-cause death was 1.1% during prasugrel treatment and 2.2% up to the end of the study. Cardiovascular death accounted for approximately three-fourths of all-cause death.

**Table 4:** Incidence of cardiovascular events

Efficacy outcomes	Cumulative incidence (%) *N* = 732	
	On treatment	Until the end of the observation period (EAS)
MACE	14 (1.9)	23 (3.1)
CV death	6 (0.8)	13 (1.8)
Non-fatal MI	5 (0.7)	5 (0.7)
Non-fatal ischemic stroke	3 (0.4)	5 (0.7)
All-cause death	8 (1.1)	17 (2.3)
Non-fatal stroke	3 (0.4)	6 (0.8)
Readmission due to angina pectoris	4 (0.5)	8 (1.1)
Revascularization	10 (1.4)	16 (2.2)
Stent thrombosis	2 (0.3)	5 (0.7)

*EAS* efficacy analysis set, *MACE* major adverse cardiovascular events, *CV* cardiovascular, *MI* myocardial infarction

## Discussion

This postmarketing observational study assessed the safety and efficacy of short-term treatment with prasugrel in patients with ACS in real-world clinical practice settings in Japan. We consider that this study provides relevant information in terms of the efficacy and safety of prasugrel as we included patients with severe cardiac disease (Killip IV) (6.7% of patients), history of ischemic stroke (5.1%), and severe renal impairment (on dialysis) (2.0%), as well as those concomitantly taking drugs that increase the tendency of bleeding AEs, such as warfarin/DOACs (2.6%) or NSAIDs (1.5%). Patients having these baseline demographics and/or taking these concomitant drugs (approximately one-sixth of the patients) were excluded from clinical studies in Japan, such as PRASFIT-ACS [8].

In approximately 99% of patients, the initial prasugrel LD and MD were 20 and 3.75 mg, respectively; 95.1% of patients received an initial LD. Prasugrel was administered as described in the package insert [10], and aspirin was used concomitantly in most patients. Nearly 70% of prasugrel treatment completions or discontinuations occurred as patients switched to other anti-platelet agents. Because prasugrel was only allowed to be prescribed for a period of 2 weeks during the first year after its launch, these patients were prescribed other anti-platelet agents for subsequent long-term treatment.

The incidence of ADRs was 8.6%, and the incidence of serious ADRs was 3.4%. The highest incidence of ADRs was for gastrointestinal disorders (3.3%). Most of the ADRs and serious ADRs were bleeding AEs. A total of 12 patients experienced major bleeding AEs. Approximately 60% (seven patients) of all major bleeding AEs were gastrointestinal disorders. This finding was consistent with the observation in a survey of clopidogrel (J-PLACE) in NSTEMI/unstable angina pectoris patients scheduled to undergo PCI [12], which suggests that gastrointestinal disorders are the main bleeding AEs in ACS patients. Therefore, preventive measures for gastrointestinal disorders might be required. Aspirin is highly likely to be a contributor to the development of these gastrointestinal disorders [13] and the prevention of low-dose aspirin-associated upper gastrointestinal injuries by proton pump inhibitors (PPIs) has been reported [14]. However, in this study, the proportion of patients receiving PPIs was less than half (47.4%) of the total population assessed. Although no significant difference was noted in the incidence of bleeding AEs between patients treated with or without concomitant PPIs in this study, concomitant use of PPIs from the start of dual anti-platelet therapy seems essential for preventing gastrointestinal disorders, especially in high-risk patients.

The incidence of major bleeding AEs in patients treated with prasugrel was 1.9% in PRASFIT-ACS [8], and was slightly lower in this study (1.6%). Furthermore, the incidences of minor bleeding AEs, clinically relevant bleeding AEs, other bleeding AEs, and all bleeding AEs were all lower in this study in comparison with PRASFIT-ACS [8]. One possible explanation for these differences is that, in this study, intraoperative bleeding of the expected amount associated with invasive procedures, such as PCI, was not reported as an AE. Another possible explanation is that the observation period in this study differed from that in PRASFIT-ACS [8].

Notably, the incidence of bleeding AEs in this study was significantly higher in female patients and patients with severe cardiovascular disease (Killip Class III or IV), in addition to patients aged 75 years or older, patients with low body weight (50 kg or less), and patients with severe renal impairment. For the elderly, patients with low body weight, and patients with severe renal impairment, prasugrel treatment should be administered with caution as specified in the “Careful Administration” section of the package insert. Women generally have lower body weight than men, and there are differences in skin tissue structure between the sexes. Therefore, the fact that a higher proportion of female patients reported subcutaneous hemorrhage is likely to be related to these observed sex differences. In fact, the second most common bleeding AE was general disorders and administration site conditions (e.g., puncture site hemorrhage) (1.0%); thus, measures to prevent puncture site bleeding might also be required. Results of the MATRIX Access study [15], a clinical trial conducted in European ACS patients who were about to undergo CAG and PCI, suggested that radial access compared with femoral access decreased the net AEs through a reduction in major bleeding AEs and death. Furthermore, in PRASFIT-ACS, the incidence of puncture site bleeding during PCI was lower in the radial access route group than in the femoral access route group [11]. In this study, there was no difference in the number of patients with puncture site bleeding AEs between groups undergoing PCI via different puncture sites, which was likely because the incidence of patients with puncture site bleeding AEs was low [1.0% (7/732)], even though it was the second most common bleeding AE. The reason for the low incidence of puncture site bleeding AEs may be that—in contrast with PRASFIT-ACS—the proportion of patients undergoing PCI via femoral access was lower (43.0%) than that undergoing PCI via radial access (51.1%). Therefore, radial access seems more appropriate for preventing puncture site bleeding. In this study, the timing of the LD did not appear to significantly affect the incidence of bleeding AEs.

Though the overall incidences of bleeding AEs were lower in this study in comparison with PRASFIT-ACS [8], after calculating the total risk score in association with the incidence of bleeding AEs, we found that the risk of bleeding increased if patients had four or all five risk factors: “female sex”, “age of 75 years or older”, “body weight of 50 kg or less”, “severe cardiovascular disease”, and “severe renal impairment”. Another study assessed the risk of bleeding in patients with ACS undergoing PCI abroad; these investigators concluded that patients with ACS have marked variability in the risk of bleeding according to sex, age, and serum creatinine, among other factors [16]. A study by Saito et al. [11], which examined periprocedural bleeding in relation to the access route for PCI in a Japanese sample, found that sex, body weight, and age were risk factors, observations that are in line with our findings. There is a possibility that the risk of bleeding will increase in the above-mentioned patients. However, an analysis adjusting for confounding effects on each risk factor was not performed. Furthermore, as a limited number of patients with severe cardiac dysfunction were evaluated in clinical trials (these patients were generally excluded), these patients will be evaluated in the ongoing PRASFIT-Practice II study, a long-term observational study in patients with ischemic heart disease.

The incidence of MACE was lower in the current study (3.1%) than in PRASFIT-ACS [8] (9.4%). Conversely, the incidences of cardiovascular death (1.8%), all-cause death (2.2%), and non-fatal ischemic stroke (0.7%) were slightly higher in the current study than those in PRASFIT-ACS [8] (1.3, 1.8, and 0.4%, respectively). The explanation for these differences may involve: (1) the difference in the duration of the observation period in each study, and (2) that PRASFIT-Practice I was an observational study reflecting the clinical use of prasugrel in a real-world setting. Thus, patients with severe conditions were included, whereas in the PRASFIT-ACS [8], such cases were excluded. The incidence of non-fatal MI was low in this study, which may also explain the low incidence of MACE compared with PRASFIT-ACS [8]. A possible reason for this may be that naturally occurring MI as well as events judged according to CAG findings and markers of myocardial injury, including creatine kinase-MB, were evaluated in PRASFIT-ACS [8]; however, only cases of MI reported by investigators under the actual conditions of use were evaluated as events in this study.

This study had several limitations. Because the study was designed as a postmarketing observational study, only patients treated with prasugrel were evaluated. As this study aimed to assess the real clinical situation in Japan, patients were not subjected to strict exclusion criteria. The observation and follow-up periods varied for each patient. As this was a short-term study, the results are only applicable to patients treated during a short period. The length of the observational period was insufficient to collect an adequate number of cardiovascular events to thoroughly evaluate safety, especially in terms of risk factors. However, the long-term observational study “PRASFIT-Practice II” will address these issues.

## Conclusion

Based on the results of this short-term clinical study in patients with ACS in a real-world acute setting, prasugrel administration at an LD of 20 mg and MD of 3.75 mg/day was considered to be acceptable for Japanese patients in terms of safety and efficacy.

## Electronic supplementary material

Below is the link to the electronic supplementary material.

Supplementary material 1 (DOCX 19 kb)
