# Supplementary material for: Prasugrel for Japanese patients with acute coronary syndrome in short-term clinical practice (PRASFIT-Practice I): a postmarketing observational study
Source: Cardiovasc Interv Ther. 2017 Feb 17;33(2):135–45. doi: 10.1007/s12928-017-0459-8 (PMC5880844; doi:10.1007/s12928-017-0459-8)
Supplement: Supplementary file 1 — Supplementary material 1 (DOCX 19 kb) [file 12928_2017_459_MOESM1_ESM.docx]

**Electronic Supplement 1**

**Definition of bleeding events**

| (1) Major bleeding (TIMI criteria) |
| --- |
| Documented intracranial hemorrhage or clinically overt bleeding with a decrease of hemoglobin by ≥5 g/dL.  Transfusion of 2 units of red cells (1 unit = 200 mL) will be converted to an increase in hemoglobin of 1 g/dL. |
| (2) Minor bleeding (TIMI criteria) |
| Documented clinically overt bleeding with a decrease of hemoglobin by ≥3 g/dL and <5 g/dL.  Transfusion of 2 units of red cells (1 unit = 200 mL) will be converted to an increase in hemoglobin of 1 g/dL. |
| (3) Clinically relevant non-major bleeding |
| Clinically relevant non-major bleeding that meets any of the following definitions:  1) Bleeding that involves a critical organ (retroperitoneal, pericardial sac, posterior chamber of the eye (e.g., vitreous bleeding and retinal bleeding), intraspinal, and intra-articular hemorrhage.  2) Gastrointestinal hemorrhage with a decrease of hemoglobin (unrelated to intubation or placement of a nasogastric tube).  3) Macroscopic hematuria without involvement of external factors.  4) Epistaxis requiring an otological procedure.  5) Gingival bleeding requiring a dental procedure.  6) Bleeding that requires termination or suspension of prasugrel treatment in the investigator’s opinion. |
| (4) Other bleeding |
| Any other bleeding event that does not fit the criteria for major bleeding, minor bleeding, and clinically relevant non-major bleeding (except intraoperative bleeding of the expected amount associated with invasive procedures such as PCI). |

TIMI, thrombolysis in myocardial infarction; PCI, percutaneous coronary intervention

**Definitions of cardiovascular events**

| (1) Death |
| --- |
| Any deaths, irrespective of etiology.  Cardiovascular death: Patients dying from cardiovascular disease.  Non-cardiovascular death: Patients dying from any cause other than cardiovascular disease. |
| (2) Non-fatal myocardial infarction |
| Symptoms suspected to be due to new-onset (acute) or recurrent myocardial infarction occur after initial PCI or CABG has been performed, and non-fatal myocardial infarction is diagnosed on the basis of myocardial injury markers (cardiac enzymes) and/or ECG findings.*  *New or recurrent ST segment deviation by ≥±1 mm or (0.1 mV) or new appearance of aberrant Q waves. |
| (3) Re-hospitalization due to angina pectoris |
| Re-hospitalization or prolongation of the hospital stay due to new-onset or recurrent chest pain, with new or recurrent ST segment deviation by ≥±1 mm or (0.1 mV). |
| (4) Cerebral stroke |
| New occurrence of neurological symptoms/signs with CT or MRI documentation of a relevant lesion. Stroke is classified as ischemic or non-ischemic stroke.  Ischemic stroke: Atherothrombotic cerebral infarction, cardiogenic cerebral embolism, lacunar infarction, and unspecified cerebral infarction  Non-ischemic stroke: Cerebral hemorrhage (including subarachnoid hemorrhage) |
| (5) Stent thrombosis |
| “Definite”, “probable”, or “possible” stent thrombosis according to the ARC definitions. |
| (6) Revascularization |
| New non-elective (emergency) PCI or CABG other than the initial PCI/CABG or as coronary thrombolytic therapy. |

PCI, percutaneous coronary intervention; CABG, coronary artery bypass grafting; ECG, electrocardiogram; CT, computed tomography; MRI, magnetic resonance imaging; ARC, Academic Research Consortium

**Electronic Supplement 2.** Summary of adverse drug reactions and serious adverse drug reactions

| **Item** | **Result** | | | |
| --- | --- | --- | --- | --- |
| **No. of patients in the safety analysis set** | 732 | | | |
| **No. of patients with ADRs** | 63 | | | |
| **No. of patients with serious ADRs** | 25 | | | |
| **Incidence of patients with ADRs (%)** | 8.6 | | | |
| **Incidence of patients with serious ADRs (%)** | 3.4 | | | |
| **Type of ADR** | **No. of patients with ADRs (%)** | | **No. of patients with serious ADRs (%)** | |
| **Blood and lymphatic system disorders** | 7 | 1.0 | 3 | 0.4 |
| **Eye disorders** | 1 | 0.1 | 1 | 0.1 |
| **Cardiac disorders** | 4 | 0.5 | 4 | 0.5 |
| **Vascular disorders** | 2 | 0.3 | 1 | 0.1 |
| **Respiratory, thoracic and mediastinal disorders** | 3 | 0.4 | 1 | 0.1 |
| **Gastrointestinal disorders** | 24 | 3.3 | 15 | 2.0 |
| **Hepatobiliary disorders** | 4 | 0.5 | 0 | 0.0 |
| **Skin and subcutaneous tissue disorders** | 7 | 1.0 | 0 | 0.0 |
| **Renal and urinary disorders** | 4 | 0.5 | 1 | 0.1 |
| **General disorders and administration site conditions** | 7 | 1.0 | 1 | 0.1 |
| **Investigations^a^** | 3 | 0.4 | 0 | 0.0 |
| **Injury, poisoning and procedural complications** | 3 | 0.4 | 0 | 0.0 |

ADRs, adverse drug reactions

^a^Abnormal laboratory findings

For System Organ Class, the number of patients with ADRs was tabulated.

MedDRA/J version 18.1.
